# Benefit of omidenepag isopropyl ophthalmic solution in treatment persistence in Japanese patients with glaucoma

**DOI:** 10.1038/s41598-025-22758-w

**Published:** 2025-11-06

**Authors:** Kenji Kashiwagi, Tadashi Nakano, Toru Nakazawa, Masahiro Fuwa, Naomi Otsuka, Daisuke Shii, Reiko Miyahara

**Affiliations:** 1 Department of Ophthalmology, University of Yamanashi, Kofu, Japan; 2 Department of Ophthalmology, The Jikei University School of Medicine, Tokyo, Japan; 3 Department of Ophthalmology, Tohoku University Graduate School of Medicine, Sendai, Japan; 4 Japan Medical Affairs Group, Santen Pharmaceutical Co., Ltd., Osaka, Japan

**Keywords:** Glaucoma, Omidenepag isopropyl ophthalmic solution, EP2 receptor agonist, Treatment persistence, Real-World study, Eye diseases, Ocular hypertension, Glaucoma

## Abstract

We conducted a retrospective observational cohort study using a nationwide claims database (JMDC database) to investigate the treatment persistence of antiglaucoma ophthalmic solution monotherapy. Patients aged 18 years or older who were diagnosed with glaucoma between November 2018 and August 2020, and prescribed omidenepag isopropyl (OMDI), prostaglandin F receptor agonists (FP), or beta-blockers (BB) monotherapies were included. A total of 36,450 patients (5,865 patients prescribed OMDI, 20,715 patients prescribed FP, and 9,870 patients prescribed BB) were analyzed. The cumulative treatment persistence rate at three years was 27.0% (95% confidence interval [CI]: 25.9–28.1) in patients prescribed OMDI, which was significantly higher than 20.9% (95% CI: 20.4–21.5) in those prescribed FP; the hazard ratio was 0.835 (95% CI: 0.806–0.865, *p* < 0.0001), calculated with the multivariate Cox proportional hazards model. The median duration of treatment persistence, calculated with the Kaplan-Meier methods, was 281 days in those prescribed OMDI, 204 days in those prescribed FP, and 152 days in those prescribed BB; the treatment persistence of OMDI tended to be longer than FP or BB. OMDI had a longer treatment persistence compared to other antiglaucoma ophthalmic solutions in the real-world setting, suggesting that OMDI would be a promising first-line treatment option in the long-term management of glaucoma.

**Supplementary Information:**

The online version contains supplementary material available at 10.1038/s41598-025-22758-w.

## Introduction

Glaucoma is a chronic and progressive eye disease that requires long-term treatment and monitoring^1–3^. Although glaucoma is the most common disease leading to visual impairment in Japan^4^, patients are often unaware of visual field abnormalities in the early stages of glaucoma, and patients are less likely to realize the importance of treatment. Patients may need to receive treatment with multiple medications for certain conditions, regularly visit the ophthalmologist, and have side effects. These can be a burden to the patients. Although treatment persistence is vital for such progressive disease, treatment persistence is generally low in patients with glaucoma^5–9^. Low treatment persistence has also been noted among Japanese patients. A real-world data study showed that approximately 40% discontinued within a year; surprisingly, nearly 30% discontinued in the first three months^10^. Along with the continuation of treatment, it is also important to maintain treatment adherence. However, the treatment adherence in glaucoma patients also remains low^11^.

Since intraocular pressure (IOP)-lowering is associated with reduced progression of visual field defect^12^, a majority of patients with glaucoma receive topical IOP-lowering medication to maintain their visual function. In Japan, as recommended in the guidelines^1^, prostaglandin F receptor agonists (FP) have been widely used as a first-line therapy for glaucoma. Despite their high efficacy and tolerability, there are some unmet clinical needs. In addition to their common ocular side effects, FP could induce distinctive local side effects, including prostaglandin-associated periorbitopathy (PAP)^13–15^, which might have a negative impact on treatment persistence. Moreover, such periorbital tissue changes could interfere with the treatment persistence due to cosmetic problems, ophthalmic surgery outcomes, and reliable IOP measurements^16,17^. Beta-blockers (BB) are also recommended for first-line therapy; however, contraindications and side effects should be taken into account^1^.

Prostaglandin E_2_ receptor EP2 subtype (EP2 receptor) agonist is also recommended as a first-line treatment option for glaucoma in Japan^1^. Omidenepag isopropyl (OMDI), the first regulatory approved EP2 receptor agonist, became available on the market in 2018 in Japan. OMDI has also been approved in several Asian countries as well as the United States. OMDI is a prodrug of omidenepag, a selective EP2 receptor agonist with a non-prostaglandin structure, which differs from that of any existing FP^18^. A pre-clinical study showed that OMDI demonstrates IOP-lowering effects by enhancing aqueous humor outflow via trabecular meshwork and uveoscleral pathways^19^, unlike FP that demonstrate IOP-lowering effects by primarily increasing uveoscleral outflow. In a clinical trial, OMDI demonstrated that its IOP-lowering effect was non-inferior to latanoprost with favorable tolerability in Japanese patients with primary open-angle glaucoma or ocular hypertension^20^. In addition, unlike FP, non-clinical and clinical studies suggest that OMDI would not cause PAP^21–25^.

As mentioned above, low persistence rates are a challenge for glaucoma treatment. Therefore, we conducted a non-interventional retrospective cohort study using a Japanese health insurance claims database provided by JMDC Inc. Tokyo, Japan (JMDC database) to investigate the treatment persistence of OMDI compared to other antiglaucoma ophthalmic solutions. The primary objective of this study was to investigate the treatment persistence rate for OMDI monotherapy compared to other antiglaucoma ophthalmic solution monotherapy, primarily FP monotherapy (i.e., tafluprost, latanoprost, travoprost, and bimatoprost). The secondary objectives were (1) to compare the treatment persistence rate for OMDI, FP, and BB monotherapy, (2) to identify the factors affecting the treatment persistence of OMDI monotherapy, (3) to identify patterns of treatment switching and use of concomitant medication after the end of treatment with OMDI monotherapy. Investigating the persistence rate of first-line glaucoma treatment in this study might provide clinically and medically meaningful insights.

## Results

### Patients

**Fig. 1:**
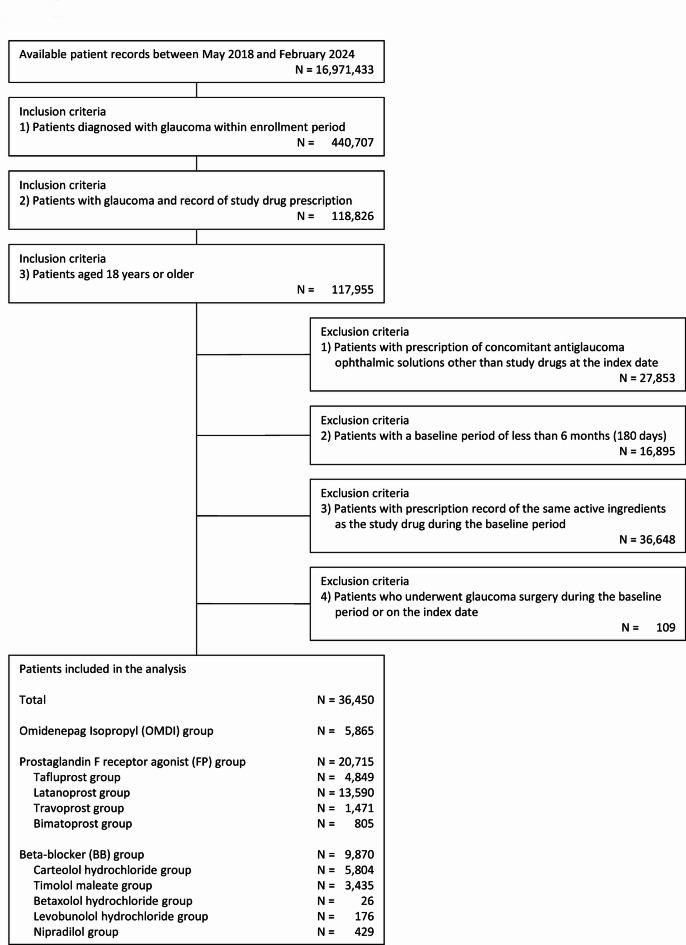
Patient selection flow.

From 16,971,433 patient records between May 2018 and February 2024 in the JMDC database, 440,707 patients who were diagnosed with glaucoma were identified. Of those, 117,955 patients met the inclusion criteria of this study. After excluding those who met the exclusion criteria, 36,450 patients (5,865 in the OMDI group, 20,715 in the FP group, and 9,870 in the BB group) were included in the analysis (Fig. 1).

The patient characteristics are summarized in Table 1. Among the overall population of 36,450 patients, 21,020 (57.7%) were male, and their mean age (± standard deviation) was 52.4 (10.25) years. The majority (35,058 patients; 96.2%) did not have a history of ocular surgery other than glaucoma surgery within six months prior to the index date. The most common type of glaucoma was primary open-angle glaucoma (11,027 patients; 30.3%). Within six months prior to the index date, 2,597 patients (7.1%) were prescribed antiglaucoma ophthalmic solution monotherapy, 467 patients (1.3%) were prescribed two antiglaucoma ophthalmic solutions, 124 patients (0.3%) were prescribed three antiglaucoma ophthalmic solutions, and 26 patients (0.1%) were prescribed four or more antiglaucoma ophthalmic solutions. A total of 30,220 patients (82.9%) were prescribed concomitant drugs other than antiglaucoma ophthalmic solutions within six months prior to the index date; of those, 28,882 patients (79.2%) were prescribed concomitant drugs other than ophthalmic solutions. The majority (30,125 patients; 82.6%) had comorbidities, such as cardiovascular diseases (3,616 patients; 9.9%), respiratory diseases (4,618 patients; 12.7%), diabetes mellitus (5,990 patients; 16.4%), psychotic disorders (3,709 patients; 10.2%), and neurological diseases (5,790 patients; 15.9%). In addition, 30,068 patients (82.5%) had ophthalmologic comorbidities other than glaucoma.

**Table 1:** Patient characteristics.

		Overall		OMDI		FP		BB	
		*n*	(%)	*n*	(%)	*n*	(%)	*n*	(%)
Total		36,450		5,865		20,715		9,870	
Sex	Male	21,020	(57.7)	2,782	(47.4)	13,007	(62.8)	5,231	(53.0)
	Female	15,430	(42.3)	3,083	(52.6)	7,708	(37.2)	4,639	(47.0)
Age category at the index date, years	18–29	878	(2.4)	124	(2.1)	404	(2.0)	350	(3.5)
	Male	530	(1.5)	66	(1.1)	252	(1.2)	212	(2.1)
	Female	348	(1.0)	58	(1.0)	152	(0.7)	138	(1.4)
	30–39	2,920	(8.0)	571	(9.7)	1,463	(7.1)	886	(9.0)
	Male	1,664	(4.6)	303	(5.2)	913	(4.4)	448	(4.5)
	Female	1,256	(3.4)	268	(4.6)	550	(2.7)	438	(4.4)
	40–49	9,461	(26.0)	1,864	(31.8)	4,942	(23.9)	2,655	(26.9)
	Male	5,200	(14.3)	834	(14.2)	3,016	(14.6)	1,350	(13.7)
	Female	4,261	(11.7)	1,030	(17.6)	1,926	(9.3)	1,305	(13.2)
	50–59	14,006	(38.4)	2,271	(38.7)	8,047	(38.8)	3,688	(37.4)
	Male	8,066	(22.1)	1,056	(18.0)	5,080	(24.5)	1,930	(19.6)
	Female	5,940	(16.3)	1,215	(20.7)	2,967	(14.3)	1,758	(17.8)
	60–69	7,718	(21.2)	922	(15.7)	4,853	(23.4)	1,943	(19.7)
	Male	4,752	(13.0)	466	(7.9)	3,169	(15.3)	1,117	(11.3)
	Female	2,966	(8.1)	456	(7.8)	1,684	(8.1)	826	(8.4)
	70–74	1,467	(4.0)	113	(1.9)	1,006	(4.9)	348	(3.5)
	Male	808	(2.2)	57	(1.0)	577	(2.8)	174	(1.8)
	Female	659	(1.8)	56	(1.0)	429	(2.1)	174	(1.8)
	Younger than median	17,430	(47.8)	3,324	(56.7)	9,106	(44.0)	5,000	(50.7)
	Male	9,680	(26.6)	1,521	(25.9)	5,561	(26.8)	2,598	(26.3)
	Female	7,750	(21.3)	1,803	(30.7)	3,545	(17.1)	2,402	(24.3)
	Median and older	19,020	(52.2)	2,541	(43.3)	11,609	(56.0)	4,870	(49.3)
	Male	11,340	(31.1)	1,261	(21.5)	7,446	(35.9)	2,633	(26.7)
	Female	7,680	(21.1)	1,280	(21.8)	4,163	(20.1)	2,237	(22.7)
	< 60	27,265	(74.8)	4,830	(82.4)	14,856	(71.7)	7,579	(76.8)
	Male	15,460	(42.4)	2,259	(38.5)	9,261	(44.7)	3,940	(39.9)
	Female	11,805	(32.4)	2,571	(43.8)	5,595	(27.0)	3,639	(36.9)
	≥ 60	9,185	(25.2)	1,035	(17.6)	5,859	(28.3)	2,291	(23.2)
	Male	5,560	(15.3)	523	(8.9)	3,746	(18.1)	1,291	(13.1)
	Female	3,625	(9.9)	512	(8.7)	2,113	(10.2)	1,000	(10.1)
Age at the index date, years	Mean	52.4		50.6		53.4		51.5	
	SD	10.25		9.61		10.12		10.68	
	Min	18		18		18		18	
	Q1	46.0		45.0		47.0		45.0	
	Median	53.0		51.0		54.0		52.0	
	Q3	60.0		57.0		60.0		59.0	
	Max	74		74		74		74	
History of ocular surgery other than glaucoma surgery within six months prior to the index date	No	35,058	(96.2)	5,802	(98.9)	19,973	(96.4)	9,283	(94.1)
	Yes	1,392	(3.8)	63	(1.1)	742	(3.6)	587	(5.9)
	Intraocular surgery	808	(2.2)	10	(0.2)	396	(1.9)	402	(4.1)
	Other than intraocular surgery	672	(1.8)	53	(0.9)	400	(1.9)	219	(2.2)
Glaucoma type at the index date	Glaucoma suspected	2,689	(7.4)	249	(4.2)	1,253	(6.0)	1,187	(12.0)
	Ocular hypertension	2,586	(7.1)	224	(3.8)	1,207	(5.8)	1,155	(11.7)
	Glaucomatous optic disc cupping	489	(1.3)	69	(1.2)	221	(1.1)	199	(2.0)
	Preperimetric glaucoma	38	(0.1)	8	(0.1)	18	(0.1)	12	(0.1)
	Primary open-angle glaucoma	11,027	(30.3)	2,020	(34.4)	6,416	(31.0)	2,591	(26.3)
	Secondary glaucoma	995	(2.7)	34	(0.6)	392	(1.9)	569	(5.8)
	Unspecified glaucoma	21,739	(59.6)	3,562	(60.7)	12,654	(61.1)	5,523	(56.0)
Hospital size (number of beds) at the index date	≤ 19	30,554	(83.8)	5,151	(87.8)	16,988	(82.0)	8,415	(85.3)
	20–99	1,005	(2.8)	164	(2.8)	588	(2.8)	253	(2.6)
	100–199	999	(2.7)	129	(2.2)	656	(3.2)	214	(2.2)
	200–299	694	(1.9)	77	(1.3)	463	(2.2)	154	(1.6)
	300–499	1,516	(4.2)	199	(3.4)	931	(4.5)	386	(3.9)
	≥ 500	1,682	(4.6)	145	(2.5)	1,089	(5.3)	448	(4.5)
Inpatient/outpatient states at the index date	Inpatient	166	(0.5)	17	(0.3)	80	(0.4)	69	(0.7)
	Outpatient	36,284	(99.5)	5,848	(99.7)	20,635	(99.6)	9,801	(99.3)
Prescription of antiglaucoma ophthalmic solutions within six months prior to the index date	No	33,236	(91.2)	4,930	(84.1)	19,193	(92.7)	9,113	(92.3)
	Yes	3,214	(8.8)	935	(15.9)	1,522	(7.3)	757	(7.7)
	CAI	256	(0.7)	64	(1.1)	134	(0.6)	58	(0.6)
	CAI/BB combination	738	(2.0)	236	(4.0)	329	(1.6)	173	(1.8)
	CAI/α2 agonist combination	1	(0.0)	1	(0.0)	0	(0.0)	0	(0.0)
	EP2	1	(0.0)	0	(0.0)	0	(0.0)	1	(0.0)
	FP	577	(1.6)	202	(3.4)	257	(1.2)	118	(1.2)
	FP/BB combination	788	(2.2)	224	(3.8)	406	(2.0)	158	(1.6)
	ROCK/α2 agonist combination	0	(0.0)	0	(0.0)	0	(0.0)	0	(0.0)
	ROCK inhibitor	223	(0.6)	32	(0.5)	134	(0.6)	57	(0.6)
	α1 inhibitor	82	(0.2)	15	(0.3)	38	(0.2)	29	(0.3)
	α2 agonist /BB combination	9	(0.0)	3	(0.1)	4	(0.0)	2	(0.0)
	α2 agonist	516	(1.4)	151	(2.6)	242	(1.2)	123	(1.2)
	BB	289	(0.8)	83	(1.4)	142	(0.7)	64	(0.6)
	Ion channel opener	326	(0.9)	90	(1.5)	151	(0.7)	85	(0.9)
	Parasympathomimetic	63	(0.2)	12	(0.2)	28	(0.1)	23	(0.2)
	Others	61	(0.2)	1	(0.0)	41	(0.2)	19	(0.2)
Number of prescribed antiglaucoma ophthalmic solutions within six months prior to the index date	1	2,597	(7.1)	772	(13.2)	1,196	(5.8)	629	(6.4)
	2	467	(1.3)	134	(2.3)	237	(1.1)	96	(1.0)
	3	124	(0.3)	26	(0.4)	72	(0.3)	26	(0.3)
	4 or more	26	(0.1)	3	(0.1)	17	(0.1)	6	(0.1)
Number of active ingredients of prescribed antiglaucoma ophthalmic solutions within six months prior to the index date	1	1,549	(4.2)	443	(7.6)	715	(3.5)	391	(4.0)
	2	1,206	(3.3)	377	(6.4)	553	(2.7)	276	(2.8)
	3	331	(0.9)	92	(1.6)	177	(0.9)	62	(0.6)
	4	104	(0.3)	19	(0.3)	63	(0.3)	22	(0.2)
	5 or more	24	(0.1)	4	(0.1)	14	(0.1)	6	(0.1)
Duration of antiglaucoma ophthalmic solution within six months prior to the index date	N	3,214		935		1,522		757	
	Mean	51.1		55.8		50.9		45.9	
	SD	50.51		49.48		51.68		48.87	
	Min	1		1		1		1	
	Q1	1.0		1.0		1.0		1.0	
	Median	44.0		63.0		38.0		29.0	
	Q3	92.0		98.0		92.0		87.0	
	Max	176		167		176		169	
Prescription of concomitant drugs other than antiglaucoma ophthalmic solutions within six months prior to the index date	No	6,230	(17.1)	999	(17.0)	3,534	(17.1)	1,697	(17.2)
	Yes	30,220	(82.9)	4,866	(83.0)	17,181	(82.9)	8,173	(82.8)
	Ophthalmic solution	12,748	(35.0)	2,185	(37.3)	6,722	(32.4)	3,841	(38.9)
	Other than ophthalmic solution	28,882	(79.2)	4,603	(78.5)	16,513	(79.7)	7,766	(78.7)
Comorbidities other than ophthalmic disease	No	6,325	(17.4)	1,082	(18.4)	3,479	(16.8)	1,764	(17.9)
	Yes	30,125	(82.6)	4,783	(81.6)	17,236	(83.2)	8,106	(82.1)
	Cardiovascular diseases	3,616	(9.9)	456	(7.8)	2,418	(11.7)	742	(7.5)
	Respiratory diseases	4,618	(12.7)	754	(12.9)	2,867	(13.8)	997	(10.1)
	Diabetes mellitus	5,990	(16.4)	728	(12.4)	3,731	(18.0)	1,531	(15.5)
	Psychotic disorders	3,709	(10.2)	625	(10.7)	2,121	(10.2)	963	(9.8)
	Neurological diseases	5,790	(15.9)	832	(14.2)	3,444	(16.6)	1,514	(15.3)
Ophthalmic comorbidities other than glaucoma	No	6,382	(17.5)	726	(12.4)	3,853	(18.6)	1,803	(18.3)
	Yes	30,068	(82.5)	5,139	(87.6)	16,862	(81.4)	8,067	(81.7)
Glaucoma surgery after the index date	No	36,304	(99.6)	5,836	(99.5)	20,627	(99.6)	9,841	(99.7)
	Yes	146	(0.4)	29	(0.5)	88	(0.4)	29	(0.3)
Time from the index date to glaucoma surgery, days	N	146		29		88		29	
	Mean	259.5		352.6		228.6		260.4	
	SD	284.87		274.80		284.53		284.97	
	Min	1		7		2		1	
	Q1	37.0		131.0		33.5		25.0	
	Median	124.0		321.0		88.0		124.0	
	Q3	398.0		490.0		353.5		401.0	
	Max	1,079		1,079		1,032		839	
Ocular surgery other than glaucoma surgery after the index date	No	34,904	(95.8)	5,725	(97.6)	19,801	(95.6)	9,378	(95.0)
	Yes	1,546	(4.2)	140	(2.4)	914	(4.4)	492	(5.0)
	Intraocular surgery	729	(2.0)	33	(0.6)	463	(2.2)	233	(2.4)
	Other than intraocular surgery	904	(2.5)	110	(1.9)	513	(2.5)	281	(2.8)
Interval between visits after the index date, days	N	26,068		4,675		14,835		6,558	
	Mean	61.7		57.4		63.1		61.8	
	SD	36.24		30.70		36.44		39.10	
	Min	0		0		0		0	
	Q1	34.0		34.0		35.6		32.9	
	Median	58.1		55.2		58.8		58.2	
	Q3	82.1		76.0		83.5		84.0	
	Max	779		387		497		779	
Interval between visual field tests after the index date, days	N	14,575		2,812		8,351		3,412	
	Mean	216.8		209.8		218.8		217.6	
	SD	110.58		97.24		113.05		114.55	
	Min	0		0		0		0	
	Q1	150.7		151.8		150.3		150.7	
	Median	193.6		191.8		194.0		194.4	
	Q3	261.5		251.9		263.5		265.0	
	Max	1,077		849		1073		1077	
Interval between OCT tests after the index date, days	N	12,541		2,340		7,193		3,008	
	Mean	192.5		198.9		191.1		190.7	
	SD	123.55		125.11		122.73		124.17	
	Min	0		1		0		0	
	Q1	112.0		118.3		111.0		111.0	
	Median	172.3		180.0		169.0		168.6	
	Q3	234.0		238.7		233.0		232.3	
	Max	1,074		1,054		1074		1,064	
Time from the index date to end of treatment with monotherapy, days	N	36,450		5,865		20,715		9,870	
	Mean	347.4		409.8		349.4		306.1	
	SD	393.05		408.73		392.67		379.01	
	Min	1		1		1		1	
	Q1	24.0		51.0		25.0		1.0	
	Median	168.0		232.0		169.0		120.0	
	Q3	600.0		782.0		602.0		484.0	
	Max	1,080		1,080		1,080		1,080	
Hospitalization after the index date	No	34,197	(93.8)	5,551	(94.6)	19,309	(93.2)	9,337	(94.6)
	Yes	2,253	(6.2)	314	(5.4)	1,406	(6.8)	533	(5.4)
Duration of hospitalization after the index date, days	N	2,253		314		1,406		533	
	Mean	12.4		10.6		12.8		12.5	
	SD	23.18		14.64		23.09		27.16	
	Min	1		1		1		1	
	Q1	3.0		3.0		3.0		3.0	
	Median	6.0		6.0		7.0		6.0	
	Q3	13.0		11.0		13.0		12.0	
	Max	488		120		304		488	

*The proportion was calculated based on the number of all eligible patients in each group.

OMDI, omidenepag isopropyl; FP, prostaglandin F receptor agonist; BB, beta-blocker; CAI, carbonic anhydrase inhibitor; EP2, EP2 receptor agonist; ROCK, Rho-associated protein kinase; OCT, optical coherence tomography; SD, standard deviation; Q1, 1-quantile; Q3, 3-quantile.

### Treatment persistence

**Fig. 2:**
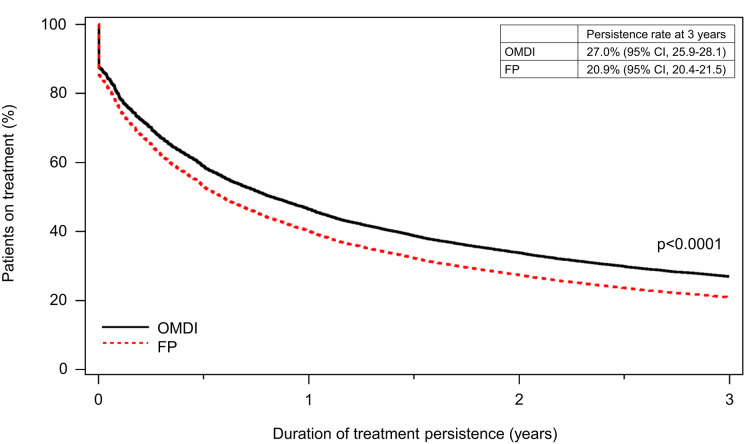
Treatment persistence of OMDI monotherapy and FP monotherapy (multivariate Cox proportional hazards model with the following four covariates: sex, age, glaucoma type, and antiglaucoma ophthalmic solutions within six months prior to the index date). OMDI, omidenepag isopropyl; FP, prostaglandin F receptor agonists; CI, confidence interval. FP included tafluprost, latanoprost, travoprost, and bimatoprost.

**Fig. 3:**
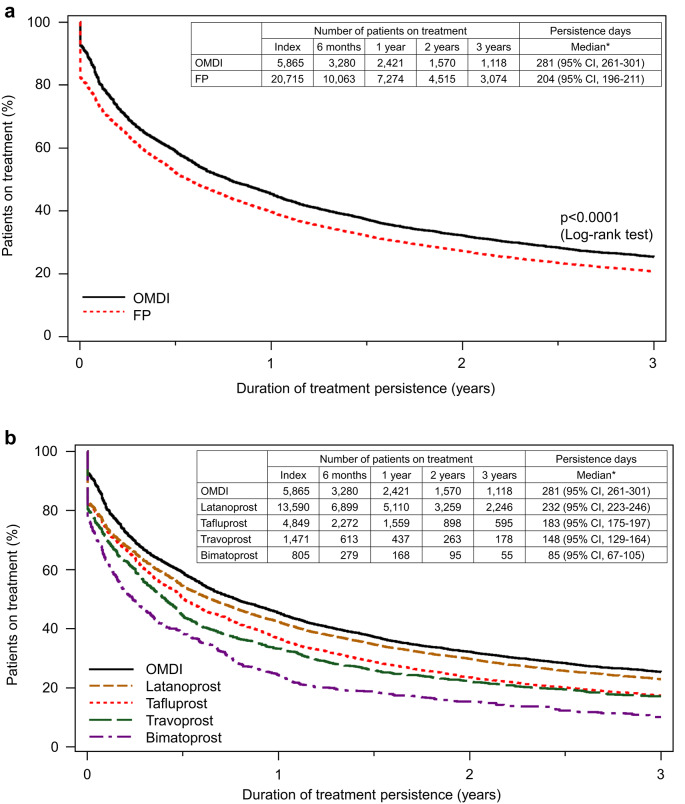
(**a**) Treatment persistence of OMDI monotherapy and FP monotherapy (Kaplan-Meier methods); (**b**) Treatment persistence of OMDI monotherapy and each individual FP monotherapy (Kaplan-Meier methods). *at the time of 50% treatment persistence rate. OMDI, omidenepag isopropyl; FP, prostaglandin F receptor agonists; CI, confidence interval. FP included tafluprost, latanoprost, travoprost, and bimatoprost.

**Fig. 4:**
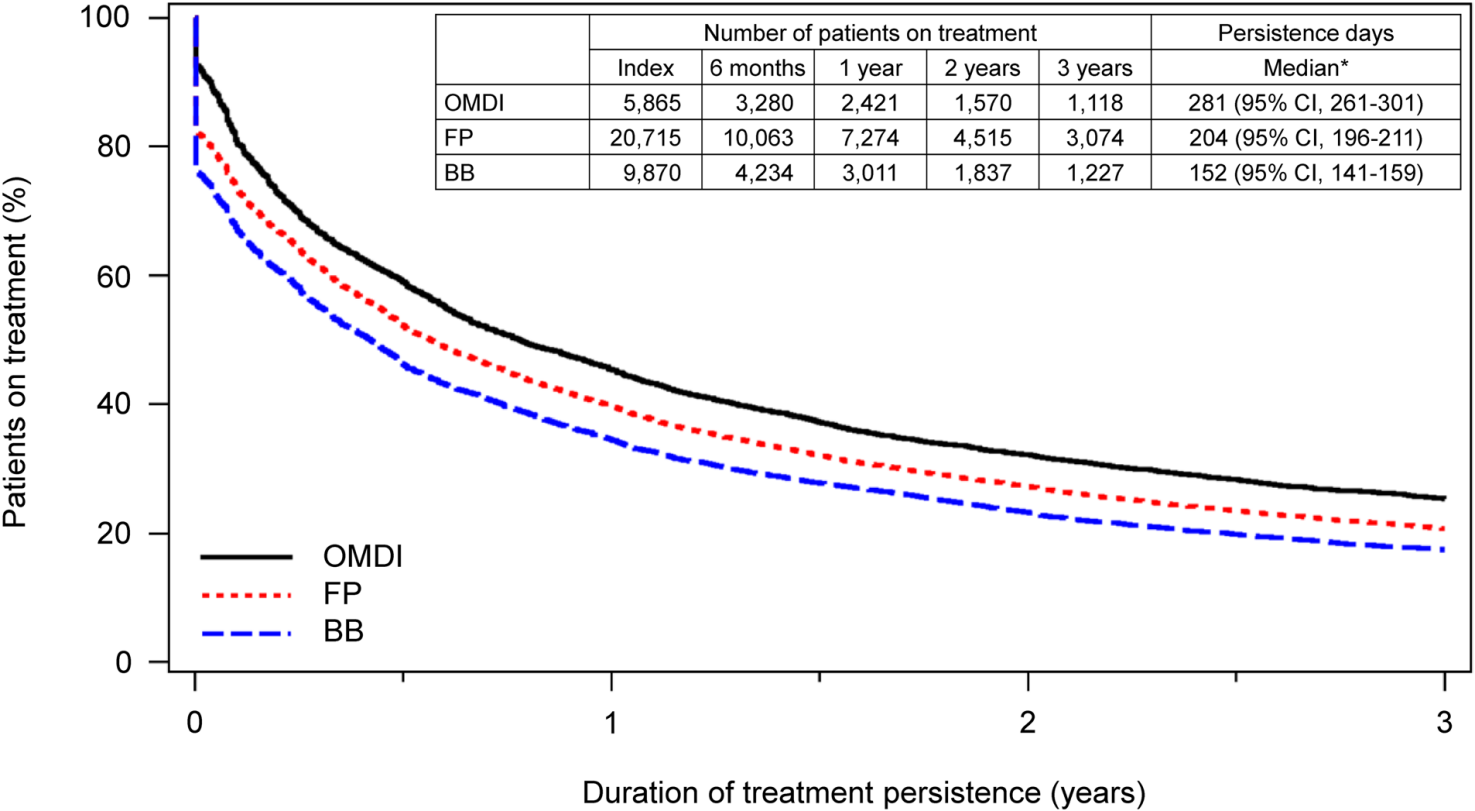
Treatment persistence of OMDI monotherapy, FP monotherapy, and BB monotherapy (Kaplan-Meier methods). *at the time of 50% treatment persistence rate. OMDI, omidenepag isopropyl; FP, prostaglandin F receptor agonists; BB, beta-blocker; CI, confidence interval. FP included tafluprost, latanoprost, travoprost, and bimatoprost. BB included carteolol hydrochloride, timolol maleate, betaxolol, levobunolol, and nipradilol.

The cumulative treatment persistence rate at three years was 27.0% (95% confidence interval [CI]: 25.9–28.1) in the OMDI group, which was significantly higher than 20.9% (95% CI: 20.4–21.5) in the FP group; the hazard ratio (HR) was 0.835 (95% CI: 0.806–0.865, *p* < 0.0001), calculated with the multivariate Cox proportional hazards model (Fig. 2). Based on the analysis using the Kaplan-Meier methods, the cumulative treatment persistence rate was 59.0% in the OMDI group and 52.2% in the FP group at six months, 45.4% and 39.7% at one year, 32.2% and 27.3% at two years, 25.4% and 20.8% at three years, respectively; moreover, the initial dropout rate was lower in the OMDI group. The median duration of treatment persistence was 281 days (95% CI: 261–301) in the OMDI group and 204 days (95% CI: 196–211) in the FP group (*p* < 0.0001, log-rank test) (Fig. 3a); the median duration of treatment persistence was 232 days (95% CI: 223–246) in the latanoprost group, 183 (95% CI: 175–197) in the tafluprost group, 148 days (95% CI: 129–164) in the travoprost group, and 85 days (95% CI: 67–105) in the bimatoprost group (Fig. 3b). In addition, the median duration of treatment persistence in the BB group was 152 days (95% CI: 141–159); the treatment persistence of OMDI tended to be longer than FP or BB (Fig. 4).

### Subsequent treatment after the end of treatment with OMDI monotherapy

**Fig. 5:**
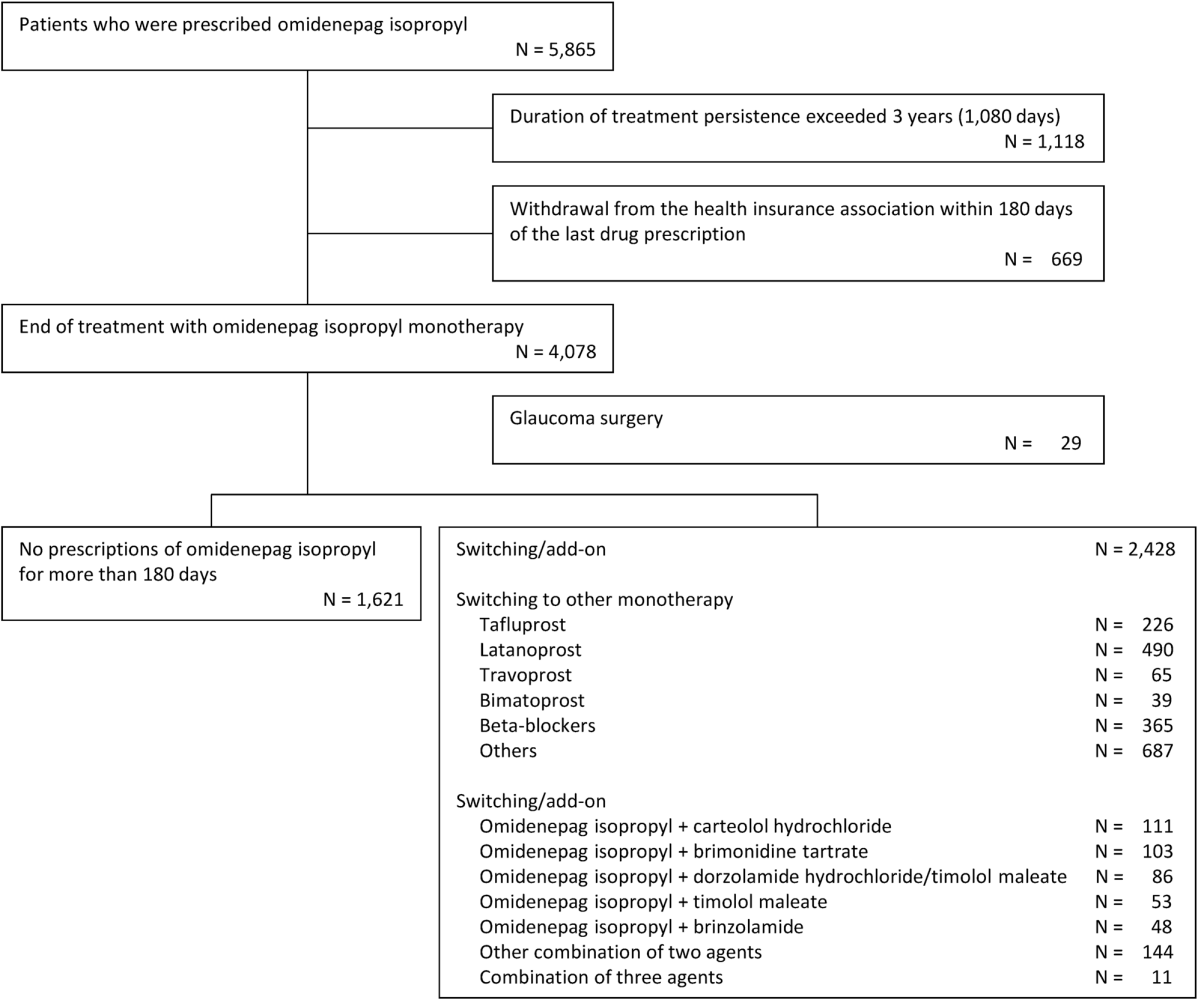
Subsequent treatment after the end of treatment with OMDI monotherapy.

Among the 4,078 patients who ended treatment with OMDI monotherapy during the study period, 29 patients underwent glaucoma surgery (88 patients for FP monotherapy and 29 for BB monotherapy, in comparison), 1,621 patients discontinued glaucoma treatment (no prescriptions of OMDI for more than 180 days), and 2,428 patients were prescribed different agents or combination treatment. Regarding the subsequent treatment, the most common FP monotherapy switched from OMDI monotherapy was latanoprost (490 patients), and the most common combination was OMDI + carteolol hydrochloride (111 patients), followed by OMDI + brimonidine tartrate (103 patients), and OMDI + dorzolamide hydrochloride/timolol maleate (86 patients) (Fig. 5).

### Multivariate analyses of factors affecting the treatment persistence of OMDI

The multivariate Cox proportional hazards model analysis identified the following variables to affect the treatment persistence of OMDI, female (HR: 0.937, 95% CI: 0.880–0.998, *p* = 0.0435), age at the index date (HR: 0.991, 95% CI: 0.988–0.994, *p* < 0.0001), diagnosis of glaucoma suspected at the index date (HR: 1.307, 95% CI: 1.124–1.520, *p* = 0.0005), secondary glaucoma diagnosis at the index date (HR: 1.867, 95% CI: 1.298–2.686, *p* = 0.0008), prescription of two antiglaucoma ophthalmic solutions within six months prior to the index date (HR: 1.504, 95% CI: 1.238–1.827, *p* < 0.0001), prescription of three antiglaucoma ophthalmic solutions within six months prior to the index date (HR: 1.717, 95% CI: 1.116–2.640, *p* = 0.0138), prescription of concomitant non-ophthalmic solutions other than antiglaucoma ophthalmic solutions within six months prior to the index date (HR: 0.887, 95% CI: 0.820–0.960, *p* = 0.0029), diabetes mellitus (HR: 1.163, 95% CI: 1.053–1.284, *p* = 0.0029), and ophthalmologic comorbidities other than glaucoma (HR: 0.882, 95% CI: 0.802–0.970, *p* = 0.0097) (Table 2). The univariate analysis data is also presented in Table S1 as supplementary data.

**Table 2:** Multivariate analyses of factors affecting the treatment persistence of OMDI.

Parameters	Reference	Categories	Hazard ratio	95% CI	*p*-values
Sex	Male	Female	0.937	0.880–0.998	0.0435
Age at the index date (1 year)	-	-	0.991	0.988–0.994	< 0.0001
Glaucoma type at the index date	POAG	Glaucoma suspected	1.307	1.124–1.520	0.0005
	POAG	Secondary glaucoma	1.867	1.298–2.686	0.0008
	POAG	Unspecified glaucoma	0.944	0.884–1.008	0.0850
Hospital size (number of beds) at the index date	≤ 19	20–99	0.893	0.742–1.076	0.2341
	≤ 19	100–199	0.984	0.800–1.210	0.8795
	≤ 19	200–299	0.824	0.616–1.103	0.1936
	≤ 19	300–499	0.969	0.820–1.146	0.7156
	≤ 19	≥ 500	1.057	0.872–1.280	0.5730
Number of prescribed antiglaucoma ophthalmic solutions within six months prior to the index date	None	1	0.983	0.896–1.078	0.7106
	None	2	1.504	1.238–1.827	< 0.0001
	None	3	1.717	1.116–2.640	0.0138
	None	≥ 4	1.250	0.312–5.009	0.7532
Prescription of concomitant ophthalmic solutions other than antiglaucoma ophthalmic solutions within six months prior to the index date	None	Yes	1.066	0.996–1.141	0.0638
Prescription of concomitant non-ophthalmic solutions other than antiglaucoma ophthalmic solutions within six months prior to the index date	None	Yes	0.887	0.820–0.960	0.0029
Cardiovascular diseases	None	Yes	0.973	0.861–1.100	0.6584
Respiratory diseases	None	Yes	1.015	0.923–1.116	0.7606
Diabetes mellitus	None	Yes	1.163	1.053–1.284	0.0029
Psychotic disorders	None	Yes	1.094	0.978–1.222	0.1151
Neurological diseases	None	Yes	0.980	0.885–1.084	0.6896
Ophthalmologic comorbidities other than glaucoma	None	Yes	0.882	0.802–0.970	0.0097

POAG, primary open-angle glaucoma; OMDI, omidenepag isopropyl; CI, confidence interval.

## Discussion

We conducted a retrospective observational study using a nationwide health insurance claims database to investigate the treatment persistence of monotherapy of first-line medications for glaucoma in Japan. To the best of our knowledge, this is the first study to compare the treatment persistence of OMDI with other antiglaucoma ophthalmic solutions, such as FP and BB, and the first report to specifically investigate the treatment persistence rate of monotherapies in a nationwide real-world setting in Japan. Our study results showed that the treatment persistence of OMDI monotherapy was significantly longer than that of FP monotherapy, and the duration of treatment persistence of OMDI monotherapy tended to be longer than that of each individual FP monotherapy and BB monotherapy. Moreover, the initial dropout rate of OMDI monotherapy was relatively low.

In Japan, a few studies have reported the treatment persistence of antiglaucoma treatment in newly diagnosed Japanese patients^10^, and Japanese patients who were prescribed combination drugs^26–28^. Those previous studies investigated the treatment persistence of initial treatment not limited to monotherapy and fixed or unfixed combination ophthalmic solutions. On the other hand, a unique feature of our study is that we investigated the treatment persistence of monotherapy. OMDI and FP are commonly used for first-line therapy for glaucoma and are often prescribed as monotherapy. Considering the long-term process of glaucoma treatment, initial therapy that can be used effectively and safely over a long period of time without the need to switch or add drugs should be selected. The Japanese guidelines state that monotherapy should be pursued^1^; thus, investigating the persistence of monotherapy in this study would be beneficial. The treatment persistence of OMDI monotherapy was significantly longer than that of FP monotherapy, moreover the initial dropout rate after initiation of OMDI monotherapy was relatively low. Since early discontinuation of the initial drug might influence the persistence of the entire glaucoma treatment, OMDI is considered to be useful as a first-line treatment in terms of the low risk of the initial dropout rate. In this study, the cumulative glaucoma treatment persistence rates for FP at 6 months, 1 year, 2 years, and 3 years were 66.5%, 58.5%, 49.5%, and 44.3%, respectively (the data are not shown), which are consistent with previous findings of 52.5% for any initial antiglaucoma ophthalmic solutions at 3 years^10^ and 45.0% for carteolol hydrochloride/latanoprost fixed-combination ophthalmic solution at 3 years^28^. This consistency ensures that the quality and reliability of the study are well-maintained. The initial dropout rate was lower in the OMDI group when analyzed using the Kaplan-Meier method. This lower initial dropout rate in the OMDI group might be influenced by the covariates (sex, age, glaucoma type, and antiglaucoma ophthalmic solutions within six months prior to the index date) incorporated into the model. Even after adjusting for these covariates, the treatment persistence of OMDI monotherapy remained significantly longer than that of FP monotherapy. Although treatment persistence has been discussed in this study, information on treatment adherence, an important factor in glaucoma management, was not available from the database; therefore, further study to investigate treatment adherence in the real-world setting is warranted.

Since the risk-benefit of a therapeutic agent is considered to have a significant impact on treatment persistence, the fact that treatment is being continued would suggest that the treatment is considered effective and tolerable for an extended period of time. The efficacy and safety of OMDI in patients with glaucoma in Japanese and Asian populations have been demonstrated in several phase III studies^20,29–31^, including those demonstrating the noninferiority of OMDI to latanoprost^20,31^. In addition, a phase III study in the US population demonstrated the noninferiority of OMDI to timolol maleate^32^. Moreover, the efficacy and safety of OMDI were demonstrated in patients who were non-/low responders to latanoprost^30,33^. Based on this evidence, OMDI is recommended as a first-line treatment option for glaucoma in Japan^1^. In addition to the favorable safety profile reported in the clinical trials^20,29^ and post-marketing observational study^34^, efficacy and safety of long-term administration of OMDI for three years in patients with normal tension glaucoma has been reported; in this study, IOP decreased over three years and visual fields were maintained^35^. Although OMDI only demonstrated non-inferiority in IOP-lowering effects in clinical trials compared to FP and BB, OMDI monotherapy had the highest treatment persistence rate and the lowest risk of the initial dropout rate among all antiglaucoma ophthalmic solutions compared in this study. In addition to lowering the IOP, it is also important to determine a feasible treatment plan for patients to improve the treatment persistence. Safety issues, including PAP, such as periorbital tissue changes causing cosmetic problems might hinder the treatment persistence. Both pre-clinical and clinical studies suggested that OMDI would not cause PAP^21–25^. Also, long-term administration of BB may have raised concerns about clinically significant adverse reactions, such as asthma and cardiovascular problems, which cannot be ruled out. The combination of the efficacy achieved together with OMDI’s different safety and tolerability profile, may have led to the differences in treatment persistence and discontinuation rates observed. Detailed data on patient-reported outcomes and the presence of PAP were not available from the database; further studies are warranted.

In this study, the proportion of female patients was relatively higher in the OMDI group than in the overall, FP, and BB groups, especially in the 40s and 50s age groups; these biases may have influenced the results for treatment persistence. Female patients might often have concerns about their cosmetic appearance. Since OMDI would not cause PAP, OMDI tended to be prescribed to those patients favorably. Taken together with our multivariate analysis, sex and age might contribute to the prolonged treatment persistence of OMDI, not only patient selection. On the other hand, OMDI was less commonly prescribed to those who had diabetes mellitus; this might be because macular edema, including cystoid macular edema, is alerted as a clinically significant adverse reaction in the OMDI package insert^36^, and physicians may have hesitated to prescribe OMDI to patients with diabetes mellitus that could be at risk of macular edema. Our multivariate analysis identified diabetes mellitus to negatively affect the treatment persistence of OMDI. As mentioned above, concerns about macular edema may have led to discontinuation of treatment with OMDI in diabetic patients before serious health issues occurred. The analysis using the multivariate Cox proportional hazards model, with the four covariates of sex, age at the index date, type of glaucoma at the index date, and the presence of prior antiglaucoma ophthalmic solutions within six months before the index date, revealed that even after adjusting for these factors, the treatment persistence of OMDI remained higher than that of FP. Taken together, our study results suggested that patients who were prescribed OMDI might have found that OMDI was more tolerable with sufficient efficacy. This robustness in treatment persistence, despite the presence of influencing factors, underscores the potential of OMDI as a preferred treatment option in clinical practice. Further studies are warranted to investigate the factors affecting treatment persistence in glaucoma treatment.

This study has several limitations. Our findings are not generalizable to the entire Japanese adult population with glaucoma since the JMDC database mostly includes health insurance data for employers of large companies, which does not include populations who were insured by other health insurance policies, such as National Health Insurance. Also, the proportion of the elderly aged 65 and over included in the database is significantly lower than that in the Japanese population because most insured employers withdraw the health insurance policies upon retirement; thus, the findings of this study may not be consistent with the general population of Japan, and the potential confounding effects of the different proportions of elderly people should be taken into consideration. In addition, the database includes data of individuals who might have provisional diagnosis temporarily given based on short-term symptoms and courses, diagnosis given for prescribing or testing purposes, and diagnosis based on the name of the disease recorded on the receipt, thus may not represent the actual health status of the patient. From JMDC database, we were unable to obtain disease severity data, which might affect the treatment persistence. The date of the last prescription of the study drug was defined as the end of treatment persistence when no study drugs were prescribed for 180 days after the index date; thus, it might not be the actual end of treatment persistence. The reasons for discontinuation or switching of treatment were unknown, as such information was not obtained from claims. With those limitations, caution should be taken in interpreting the study results, and conducting additional studies using different data sources might help to pursue better understanding of glaucoma treatment in clinical practice in Japan.

In conclusion, our study demonstrated that OMDI monotherapy exhibited longer treatment persistence compared to other antiglaucoma ophthalmic solution monotherapies in a real-world setting. This finding suggests that OMDI is a promising first-line treatment option for the long-term management of glaucoma.

## Methods

### Study design and data source

This is a retrospective observational cohort study using the JMDC database. The database is a claims database that has been accumulating receipts (inpatient, outpatient, and prescription) and health checkup data from multiple health insurance associations. The data are cumulated from approximately 17 million beneficiaries, covered under employment-based health insurance schemes. The database provides longitudinal data, and patients can be tracked even if they transfer to different medical institutions; however, patients cannot be tracked after withdrawal from the health insurance association. It has been widely used for health economics, epidemiology, and outcomes research. It is one of the most comprehensive real-world healthcare data resources available in Japan; its nationwide coverage represents the general population in various aspects, and real-world study data obtained from such database cannot be obtained from clinical trials or Japan glaucoma society-centered studies by glaucoma specialists.

### Study population

Inclusion criteria were patients aged 18 years or older who were diagnosed with glaucoma between November 2018 and August 2020 (enrollment period), and prescribed OMDI monotherapy, FP monotherapy, or BB monotherapy (study drugs) at the index date (defined as the earliest prescription date of any of the above study drugs), and whose data were available May 2018 and February 2024 (study period). Patients with glaucoma were identified using the International Statistical Classification of Diseases and Related Health Problems, 10th Revision (ICD-10) codes of H40. Baseline period was defined as the period of six months prior to the index date; the follow-up period was a maximum of three years after the index date. Patients with prescriptions of concomitant antiglaucoma ophthalmic solutions other than study drugs at the index date, those with a baseline period of less than 6 months (180 days), those with a prescription record of the same active ingredients as the study drug during the baseline period, or those who underwent glaucoma surgery during the baseline period or the index date were excluded.

### Outcomes and variables

The primary outcome was the treatment persistence rate for OMDI monotherapy compared to FP monotherapy (i.e., tafluprost, latanoprost, travoprost, and bimatoprost). The secondary outcomes included the treatment persistence rate for OMDI monotherapy compared to FP and BB monotherapy (BB includes both once-daily and twice-daily formulations), the factors affecting the treatment persistence of OMDI, and the number and type of antiglaucoma ophthalmic solutions (combination agent was counted as one agent) after the end of treatment with OMDI monotherapy. The end of treatment persistence was defined as the last prescription date of the study drugs, with no study drugs prescribed for 180 days after the index date, or one day prior to the date the study drugs were switched to another agent or combination of multiple agents. If glaucoma surgery was performed after the index date, the day before the glaucoma surgery was considered as the end of treatment persistence. The last drug prescription date was considered the censored date if the patient withdrew from the health insurance association within 180 days of the last drug prescription. Since the maximum follow-up period for this study was set at 3 years, the duration of treatment persistence was considered as 1,080 days when the duration of treatment persistence exceeded 3 years.

The following variables regarding the patient characteristics were identified: sex, age at the index date, history of ocular surgery (other than glaucoma surgery) within six months prior to the index date, glaucoma type, hospital size (number of beds), and inpatient/outpatient states at the index date, prescription of antiglaucoma ophthalmic solution and duration of their prescription within six months prior to the index date, concomitant prescription of drugs other than antiglaucoma ophthalmic solution within six months prior to the index date, and comorbidities. In addition, information on ocular surgery, interval of visits and eye examinations, treatment persistence, and hospitalization after the index date was also collected. If the date of the prescription or glaucoma surgery was not documented, it was supplemented with the 15th of the month in which the receipt was issued.

### Data analysis

A preliminary assessment revealed that ~ 5,900 patients with a prescription of OMDI monotherapy and ~ 20,000 patients with a prescription of FP monotherapy between November 2018 and June 2020. The primary outcome of this study was treatment persistence; as the primary analysis was performed using a Cox proportional hazards model, the log-rank test was used to determine sample size. Since there were no reports on treatment persistence rates for any study drugs using the receipt database in Japan, we set the minimum difference in treatment persistence rates between the OMDI group and FP group to 5%, and detected differences at median duration of the treatment persistence with HR of 0.868 and statistical power of 80%. The sample size calculation showed that the required number of patients was 1,653; thus, the number of patients necessary to detect differences could be achieved in this study.

For the primary outcome, the treatment persistence, the HR and its 95% confidence interval, and p-values for the OMDI group relative to the FP group were calculated using a multivariate Cox proportional hazards model with the following covariates: sex, age, glaucoma type, and antiglaucoma ophthalmic solutions within six months prior to the index date. The treatment persistence rate was estimated by Kaplan-Meier methods and log-rank test was used for comparison. Multivariate Cox proportional hazards model was used for identifying factors affecting treatment persistence of OMDI monotherapy. Statistical analyses were performed using Amazon Redshift and Statistical Analysis Software (SAS version 9.4), and p-values < 0.05 were considered to be statistically significant.

### Ethical statement

The study design was approved by the Kyoyukai RiverSide Clinic Institutional Review Board, Sapporo, Japan (ID: RSC-2407RB01, approved on July 30, 2024). This study is registered with UMIN-CTR (UMIN000055230). As this study involves data that exist in an anonymized structured format and contains no personal information, obtaining informed consent from subjects was not required; using only un-linkable anonymized data is outside the scope of the “Ethical Guidelines for Medical and Biological Research Involving Human Subjects” set by the Japanese government.

## Supplementary Information

Below is the link to the electronic supplementary material.

Supplementary Material 1

## Data Availability

The data supporting the findings in this study are from JMDC Inc., but there are restrictions on the availability of the data as these were used under license for the present study. Requests for access to the data should be directed to the corresponding author.
